# Prevalence and Associated Factors of Diabetes and Impaired Fasting Glucose in Chinese Hypertensive Adults Aged 45 to 75 Years

**DOI:** 10.1371/journal.pone.0042538

**Published:** 2012-08-03

**Authors:** Xianhui Qin, Jianping Li, Yan Zhang, Wei Ma, Fangfang Fan, Binyan Wang, Houxun Xing, Genfu Tang, Xiaobin Wang, Xin Xu, Xiping Xu, Yong Huo

**Affiliations:** 1 Institute for Biomedicine, Anhui Medical University, Hefei, China; 2 Department of Cardiology, Peking University First Hospital, Beijing, China; 3 Lianyungang Center for Advanced Research in Cardiovascular Diseases, Lianyungang, China; 4 School of Health Administration, Anhui Medical University, Hefei, China; 5 Department of Population, Family and Reproductive Health, Center on the Early Life Origins of Disease, Johns Hopkins University Bloomberg School of Public Health, Baltimore, Maryland, United States of America; 6 Institute of Nephrology, Southern Medical University, Guangzhou, China; National Institutes of Health - National Institute of Child Health and Human Development, United States of America

## Abstract

**Objective:**

This study examined the prevalence of impaired fasting glucose (IFG) and diabetes and their associated factors in 17,184 Chinese hypertensive adults aged 45–75 years.

**Methods:**

A cross-sectional investigation was carried out in a rural area of Lianyungang, China. Previously undiagnosed diabetes [fasting plasma glucose (FPG) ≥7.0mmol/l] and IFG (6.1–6.9mmol/l) were defined based on FPG concentration. Previously diagnosed diabetes was determined on the basis of self-report. Total diabetes included both previously diagnosed diabetes and previously undiagnosed diabetes.

**Results:**

The prevalence of previously diagnosed diabetes, undiagnosed diabetes, and IFG were 3.4%, 9.8%, and 14.1%, respectively. About 74.2% of the participants with diabetes had not previously been diagnosed. In the multivariable logistic-regression model, older age, men, antihypertensive treatment, obesity (BMI ≥25kg/m^2^), abdominal obesity (waist circumference ≥90cm for men and ≥80cm for women), non-current smoking, a family history of diabetes, higher heart rate, lower physical activity levels, and inland residence (versus coastal) were significantly associated with both total diabetes and previously undiagnosed diabetes. Furthermore, methylene- tetrahydrofolate reductase (MTHFR) 677 TT genotype was an independent associated factor for total diabetes, and current alcohol drinking was an independent associated factor for previously undiagnosed diabetes. At the same time, older age, men, abdominal obesity, non-current smoking, current alcohol drinking, a family history of diabetes, higher heart rate, and inland residence (versus coastal) were important independent associated factors for IFG.

**Conclusion:**

In conclusion, we found a high prevalence of diabetes in Chinese hypertensive adults. Furthermore, about three out of every four diabetic adults were undiagnosed. Our results suggest that population-level measures aimed at the prevention, identification (even if only based on the FPG evaluation), and treatment of diabetes should be urgently taken to overcome the diabetes epidemic in Chinese hypertensive adults.

## Introduction

Cardiovascular disease (CVD) has become the leading cause of death in China. Diabetes confers about a two-fold excess risk for coronary heart disease, major stroke subtypes, and deaths attributed to other vascular causes, independently from other conventional risk factors [1]. The prevalence of diabetes is high and is increasing in China [2], [3]. The prevalence of diabetes was 9.7% and 4.9%, respectively, in the cross-sectional survey of a nationally representative sample of Chinese adults from June 2007 through May 2008 (46,239 adults, 20 years of age or older) [4] and from January 2007 through October 2010 (47,204 adults, 18 years of age or older) [5]. Hypertension is also one of the most important modifiable risk factors for CVD, and is the leading cause of death worldwide [6]. The prevalence of hypertension was about 27.2% in 2000 in China [7]. Most importantly, diabetes and hypertension, having insulin resistance as a possible common background factor, are often associated [8], and the frequencies of cerebrovascular disease and ischemic heart disease are known to be markedly elevated by the co-existence of hypertension and diabetes [9]. Therefore, prevention and control of diabetes is important for the prevention and treatment of CVD in a hypertensive population.

It has been suggested that many of the established risk factors, including hypertension and diabetes, have both genetic and environmental components. Methylenetetrahydrofolate reductase (MTHFR) is the main regulatory enzyme for homocysteine metabolism. Previous studies found that the MTHFR 677C→T variant was associated with an increased risk of hypertension [10] and stroke [11]. Furthermore, the prevalence of MTHFR 677TT genotype was about 25% in a previous hypertensive adult study in different Chinese regions [12], [13]. However, the relationship of MTHFR C677T polymorphism with diabetes is still inconclusive.

The prevalence of diabetes and impaired fasting glucose (IFG) varies significantly around the world [14]. Therefore, planning for health plans and preventive measures requires necessary information from the field of diabetes and IFG that is specific for different geographical areas. However, to our knowledge, no previous publication has studied the prevalence of diabetes and IFG in Chinese hypertensive adults in coastal areas. For this reason, the present study examined the prevalence of diabetes and IFG and their associated factors, including MTHFR C677T polymorphism, in Chinese hypertensive adults aged 45–75 years in Lianyungang, China.

## Methods

### Study Site

Lianyungang, Jiangsu province, China, is located on the shore of the Huang Hai (between 118°24′ and 119°48′ east longitude and 34°11′ and 35°07′ north latitude). Currently, the city governs three districts (Xinpu, Lianyun and Haizhou) and four counties (Ganyu, Donghai, Guanyun and Guannan). Lianyungang is one of the first 14 Chinese coastal cities to open to the outside world, and has experienced rapid economic development in recent years.

### Study Population

The study subjects were participants of an ongoing China Stroke Primary Prevention Trial (CSPPT). CSPPT is a multi-center randomized controlled trial designed to confirm that enalapril maleate and folic acid tablets combined is more effective in preventing stroke among patients with hypertension when compared with enalapril maleate alone. Details regarding inclusion/exclusion criteria, treatment assignment, and outcome measures of the trial have been described elsewhere (http://clinicaltrials.gov/ct2/show/NCT00794885). In the current study, we included subjects from Lianyungang who participated in the screening phase of the CSPPT.

Briefly, we conducted a community-based screening in 20 townships within two counties (Ganyu, which is coastal, and Donghai, which is inland) in Lianyungang of Jiangsu province, East China, from October 2008 to September 2009. The inclusion criteria were as follows: 1) aged 45–75 years; and 2) seated systolic blood pressure (SBP) ≥140 mmHg and/or seated diastolic blood pressure (DBP) ≥90 mmHg in both of two screening visits (with at least 24 hours between visits) or currently under anti-hypertension treatment. Participants were excluded if they reported a history of myocardial infarction, stroke, heart failure, cancer, and/or serious mental disorders; or if they were unwilling to participate in the survey. This study was approved by the Ethics Committee of the Institute of Biomedicine, Anhui Medical University, Hefei, China. Written informed consent was obtained from each participant before data collection.

### Data Collection Procedures

Baseline data collection was conducted by trained research staff according to the standard operating procedure. Each participant was interviewed using a standardized questionnaire designed specifically for this study. The question about standard of living was phrased as follows, “How does your standard of living compare to others?” and a choice of three responses: bad, medium, and good was provided. The question about physical activity was phrased as follows, “How do you describe your daily physical activity level?” and a choice of three responses: low, moderate, and high was provided. Finally, the question regarding family history was phrased as follows, “Has any of your immediate family (mother, father and/or siblings) had any of the following conditions?”, and the choices of hypertension, diabetes, coronary heart disease (CHD) and stroke were given.

Anthropometric measurements, including height, weight and waist circumference were taken using the standard operating procedure. Height was measured without shoes to the nearest 0.1 cm on a portable stadiometer. Weight was measured in light indoor clothing without shoes to the nearest 0.1 kg. Body mass index (BMI) was calculated as weight (kilograms)/height (meters) squared. Waist circumference (WC) was measured as the minimum circumference between the inferior margin of the ribcage and the crest of the ileum [15], [16].

Seated blood pressure (BP) measurements were obtained by trained research staff after subjects had been seated for 10 minutes using a mercury manometer with the standard method of calibration and appropriately sized cuffs, according to the standard operating procedure. Triplicate measurements on the same arm were taken, with at least 2 minutes between readings. Resting heart rates were measured by pulse palpation (at least 30 sec.) after the third measurement [17]. Each patient’s systolic and diastolic blood pressures were calculated as the mean of the three independent measures. Blood pressure measured at visit 2 was used for analysis.

### Blood Sample Collection and Laboratory Methods

After 12–15 hours of fasting, a venous blood sample was obtained from each subject. Serum or plasma samples were separated within 30 min. of collection and were stored at −70°C, which were used for measurement of glucose concentrations using a Dade Dimension Chemistry Analyzer (Siemens, Germany). DNA was extracted from leukocytes in peripheral blood using standard techniques. MTHFR C677T genotype was determined by Taqman assay designed and manufactured by Applied Biosystems (Foster City, CA).

### Statistical Analysis

Hypertension was categorized into 3 grades: grade 1, SBP 140–159 and/or DBP 90–99 mmHg; grade 2, SBP 160–179 and/or DBP 100–109 mmHg; grade 3, SBP≥180 and/or DBP≥110 mmHg. Treated hypertension was defined as receiving antihypertensive medication within the past 2****weeks. Current smoking was defined as having smoked at least 1 cigarette per day or ≥18 packs in the last year. Current drinking was defined as drinking alcohol at least 2 times per week in the last year. Obesity was defined as a BMI of ≥25kg/m^2^ [18]. Abdominal obesity was defined according to the guidelines of the International Diabetes Federation for Chinese populations as a waist circumference ≥90 cm for men and ≥80 cm for women [19]. Previously undiagnosed diabetes [fasting plasma glucose (FPG) ≥7.0mmol/l] and IFG (6.1–6.9mmol/l) were defined using World Health Organization criteria [20] based on FPG concentration. Previously diagnosed diabetes was identified by a positive response to the question “Has a doctor ever told you that you had diabetes?” Total diabetes included both previously diagnosed diabetes and previously undiagnosed diabetes.

Means and proportions were calculated for population characteristics according to FPG categories (FPG<6.1mmol/l, IFG, previously undiagnosed diabetes, and previously diagnosed diabetes). The differences in population characteristics were compared using one-way analysis of variance or chi-square test. The adjusted odds ratios and 95% confidence interval (CI) of having IFG, previously undiagnosed diabetes and total diabetes were determined from multivariable logistic-regression models that included age group (45 to 54, 55 to 64, and 65 to 75 years), sex, cigarette smoking, alcohol drinking, antihypertensive treatment status (treated and untreated), hypertension grades (controlled blood pressure or grade 1 hypertension, grade 2 hypertension, or grade 3 hypertension), obesity, abdominal obesity, heart rate (<80, 80–100, or ≥100 beats/min), MTHFR C677T polymorphism, geographic region (coastal or inland), standard of living (bad, medium or good), education level (illiterate, primary level, elementary or higher levels), physical activity level (low, moderate, high) and family history of hypertension, diabetes, CHD or stroke. All of the statistical analyses were performed in SAS 8.2 (SAS Institute, Cary, NC, USA).

## Results

Overall, 19,705 participants aged 45–75 years with hypertension were screened. In this report, study participants with CVD (n = 604), cancer (n = 46), dyslipidemia (n = 519), or with any missing data (n = 1088) regarding antihypertensive treatment status, age, sex, height, weight, WC, smoking status, drinking status, standard of living, education and physical activity levels, reported diabetes status, MTHFR C677T polymorphism, heart rate, FPG, and family history of hypertension, coronary heart disease, diabetes and stroke were excluded. Our final analysis included 17,184 participants.

The prevalence rates of previously diagnosed diabetes, undiagnosed diabetes, and IFG were 3.4%, 9.8%, and 14.1%, respectively. About 74.2% of the participants with diabetes had not previously been diagnosed. In general, participants with previously diagnosed diabetes had the highest fasting plasma glucose levels, and were older, more likely to have a family history of diabetes, more likely to take antihypertensive treatment, less likely to smoke cigarettes and drink alcohol, and less likely to participate in physical activity (Table 1).

**Table 1:** Characteristics of study participants according to plasma glucose categories, *n* (%).

	*Total*	*FPG<6.1* *mmol/L*	*IFG*	*Previously* *undiagnosed* *diabetes*	*Previously* *diagnosed* *diabetes*	*P value*
N	17184	12492	2430	1679	583	
Age (y)^1^	59.6(7.6)	59.3(7.6)	60.0(7.6)	60.1(7.4)	61.0(7.0)	<0.001
Age group (y)						
45–54	5157(30.0)	3928(31.4)	667(27.4)	442(26.3)	120(20.6)	<0.001
55–64	7442(43.3)	5330(42.7)	1072(44.1)	757(45.1)	283(48.5)	
65–75	4585(26.7)	3234(25.9)	691(28.4)	480(28.6)	180(30.9)	
Sex, male	6334(36.9)	4636(37.1)	936(38.5)	602(35.9)	160(27.4)	<0.001
FPG, mmol/l^1^	5.8(1.9)	5.1(0.6)	6.5(0.3)	8.9(3.0)	9.8(3.8)	<0.001
Antihypertensive Treatment,Treated	7948(46.3)	5626(45.0)	1141(47.0)	819(48.8)	362(62.1)	<0.001
SBP (mm Hg)	168.6(20.8)	168.2(20.7)	169.6(21.3)	170.4(20.8)	167.0(20.3)	<0.001
DBP (mm Hg)	95.3(11.9)	95.6(11.8)	95.4(12.2)	95.1(12.1)	90.2(11.0)	<0.001
HTN Grades						
Controlled BP or Grade 1^2^	4810(28.0)	3528(28.2)	658(27.1)	426(25.4)	198(34.0)	<0.001
Grade 2	6930(40.3)	5069(40.6)	953(39.2)	683(40.7)	225(38.6)	
Grade 3	5444(31.7)	3895(31.2)	819(33.7)	570(33.9)	160(27.4)	
BMI (kg/m^2^)						
Mean^1^	25.6(3.6)	25.5(3.5)	25.8(3.6)	26.4(3.7)	25.9(3.4)	<0.001
Obesity^3^	9384(54.6)	6596(52.8)	1390(57.2)	1059(63.1)	339(58.1)	<0.001
Waist Circumference						
Mean(cm)^1^	85.5(9.6)	84.9(9.5)	86.3(10.0)	87.9(9.8)	87.6(9.2)	<0.001
Abdominal Obesity^4^	10276(59.8)	7181(57.5)	1510(62.1)	1157(68.9)	428(73.4)	<0.001
Current Smoking	3819(22.2)	2910(23.3)	504(20.7)	328(19.5)	77(13.2)	<0.001
Current Drinking	3807(22.2)	2786(22.3)	587(24.2)	374(22.3)	60(10.3)	<0.001
Family history of HTN	6551(38.1)	4860(38.9)	896(36.9)	585(34.8)	210(36.0)	0.003
Family history of diabetes	857(5.0)	519(4.2)	118(4.9)	124(7.4)	96(16.5)	<0.001
Family history of CHD	691(4.0)	509(4.1)	94(3.9)	55(3.3)	33(5.7)	0.082
Family history of stroke	2320(13.5)	1753(14.0)	303(12.5)	198(11.8)	66(11.3)	0.008
Heart rate (beats/min)						
Mean^1^	74.1(10.7)	73.4(10.3)	75.3(11.3)	76.7(11.6)	76.2(11.2)	<0.001
<80	12636(73.5)	9513(76.2)	1684(69.3)	1065(63.4)	374(64.2)	<0.001
80–100	4128(24.0)	2732(21.9)	668(27.5)	542(32.3)	186(31.9)	
≥100	420(2.4)	247(2.0)	78(3.2)	72(4.3)	23(3.9)	
MTHFR C677T						
CC	4049(23.6)	2974(23.8)	572(23.5)	373(22.2)	130(22.3)	0.410
CT	8593(50.0)	6263(50.1)	1210(49.8)	837(49.9)	283(48.5)	
TT	4542(26.4)	3255(26.1)	648(26.7)	469(27.9)	170(29.2)	
Counties						
Ganyu(coastal)	7477(43.5)	5639(45.1)	938(38.6)	663(39.5)	237(40.7)	<0.001
Donghai(inland)	9707(56.5)	6853(54.9)	1492(61.4)	1016(60.5)	346(59.3)	
Living Standards						
Bad	1899(11.1)	1361(10.9)	261(10.7)	203(12.1)	74(12.7)	0.087
Medium	13295(77.4)	9730(77.9)	1867(76.8)	1264(75.3)	434(74.4)	
Good	1990(11.6)	1401(11.2)	302(12.4)	212(12.6)	75(12.9)	
Education						
Illiterate	11306(65.8)	8160(65.3)	1616(66.5)	1140(67.9)	390(66.9)	0.182
Primary level	2515(14.6)	1873(15.0)	325(13.4)	237(14.1)	80(13.7)	
Elementary or higher levels	3363(19.6)	2459(19.7)	489(20.1)	302(18.0)	113(19.4)	
Physical Activity						
Low	6929(40.3)	4844(38.8)	1021(42.0)	731(43.5)	333(57.1)	<0.001
Moderate	6625(38.6)	4928(39.4)	912(37.5)	630(37.5)	155(26.6)	
High	3630(21.1)	2720(21.8)	497(20.5)	318(18.9)	95(16.3)	

BMI = body mass index, CHD = coronary heart disease, DBP = diastolic blood pressure, FPG = fasting plasma glucose, HTN = hypertension, IFG = impaired fasting glucose, MTHFR =  methylenetetrahydrofolate reductase, SBP = systolic blood pressure.

1 Means (SD);^ 2^474 subjects with antihypertensive treatment and controlled blood pressure were included;

3 Obesity was defined as a BMI of ≥25kg/m^2^; ^4^Abdominal obesity was defined as a waist circumference ≥90 cm for men and ≥80 cm for women.

The prevalence rates of MTHFR C677T polymorphisms 677 CC, 677 CT, and 677TT were 23.6%, 50.0%, and 26.4%, respectively. This population had no significant deviations in genotype distributions from expected Hardy-Weinberg equilibrium.

In the multivariable logistic-regression model, older age, men, antihypertensive treatment, obesity (BMI ≥25kg/m^2^), abdominal obesity (waist circumference ≥90cm for men and ≥80cm for women), non-current smoking, a family history of diabetes, higher heart rate, lower physical activity levels, and inland residence (versus coastal) were significantly associated with both total diabetes and previously undiagnosed diabetes. Furthermore, MTHFR 677 TT genotype was an independent associated factor for total diabetes, and current alcohol drinking was an independent associated factor for previously undiagnosed diabetes. At the same time, older age, men, abdominal obesity, non-current smoking, current alcohol drinking, a family history of diabetes, higher heart rate, and inland residence (versus coastal) were important independent associated factors for IFG (Table 2).

Similar trends were observed in men and women with a coastal or inland residence (data not shown).

**Table 2:** Adjusted^1^ odds ratios (95% confidence intervals) of having diabetes (total or previously undiagnosed) or IFG in different subgroups.

	*IFG*		*Previously undiagnosed diabetes*		*Total diabetes*	
	Prevalence,n (%)	Adjusted OR(95% CI)	Prevalence,n (%)	Adjusted OR(95% CI)	Prevalence,n (%)	Adjusted OR(95% CI)
Total	2430(14.1)		1679(9.8)		2262(13.2)	
Age (y)						
45–55	667(12.9)	1.00(ref)	442(8.6)	1.00(ref)	562(10.9)	1.00(ref)
55–65	1072(14.4)	1.17(1.05–1.31)	757(10.2)	1.20(1.06–1.37)	1040(14.0)	1.32(1.17–1.48)
65–75	691(15.1)	1.20(1.05–1.37)	480(10.5)	1.22(1.04–1.42)	660(14.4)	1.32(1.15–1.52)
Sex						
Men	936(14.8)	1.00(ref)	602(9.5)	1.00(ref)	762(12.0)	1.00(ref)
Women	1494(13.8)	0.76(0.66–0.86)	1077(9.9)	0.80(0.68–0.94)	1500(13.8)	0.82(0.71–0.95)
Antihypertensive Treatment						
Untreated	1289(14.0)	1.00(ref)	860(9.3)	1.00(ref)	1081(11.7)	1.00(ref)
Treated	1141(14.3)	1.05(0.96–1.15)	819(10.3)	1.13(1.01–1.25)	1181(14.9)	1.26(1.14–1.38)
HTN Grades						
Controlled BP or Grade 1^2^	658(13.7)	1.00(ref)	426(8.9)	1.00(ref)	624(13.0)	1.00(ref)
Grade 2	953(13.8)	1.00(0.90–1.12)	683(9.9)	1.09(0.95–1.24)	908(13.1)	0.99(0.89–1.11)
Grade 3	819(15.0)	1.09(0.97–1.22)	570(10.5)	1.13(0.98–1.29)	730(13.4)	0.99(0.88–1.12)
Obesity^3^						
No	1040(13.3)	1.00(ref)	620(7.9)	1.00(ref)	864(11.1)	1.00(ref)
Yes	1390(14.8)	1.11(0.99–1.24)	1059(11.3)	1.26(1.11–1.44)	1398(14.9)	1.13(1.01–1.26)
Abdominal Obesity ^4^						
No	920(13.3)	1.00(ref)	522(7.6)	1.00(ref)	677(9.8)	1.00(ref)
Yes	1510(14.7)	1.17(1.04–1.32)	1157(11.3)	1.48(1.28–1.71)	1585(15.4)	1.58(1.39–1.80)
Current smoking						
No	1926(14.4)	1.00(ref)	1351(10.1)	1.00(ref)	1857(13.9)	1.00(ref)
Yes	504(13.2)	0.76(0.66–0.87)	328(8.6)	0.78(0.67–0.92)	405(10.6)	0.76(0.66–0.88)
Current drinking						
No	1843(13.8)	1.00(ref)	1305(9.8)	1.00(ref)	1828(13.7)	1.00(ref)
Yes	587(15.4)	1.27(1.11–1.45)	374(9.8)	1.23(1.05–1.45)	434(11.4)	1.06(0.91–1.23)
FHH						
No	1534(14.4)	1.00(ref)	1094(10.3)	1.00(ref)	1467(13.8)	1.00(ref)
Yes	896(13.7)	0.95(0.86–1.05)	585(8.9)	0.85(0.76–0.96)	795(12.1)	0.83(0.75–0.92)
FHD						
No	2312(14.2)	1.00(ref)	1555(9.5)	1.00(ref)	2042(12.5)	1.00(ref)
Yes	118(13.8)	1.25(1.01–1.54)	124(14.5)	2.02(1.64–2.49)	220(25.7)	2.75(2.32–3.27)
FHC						
No	2336(14.2)	1.00(ref)	1624(9.8)	1.00(ref)	2174(13.2)	1.00(ref)
Yes	94(13.6)	0.99(0.79–1.25)	55(8.0)	0.81(0.60–1.08)	88(12.7)	0.92(0.72–1.17)
FHS						
No	2127(14.3)	1.00(ref)	1481(10.0)	1.00(ref)	1998(13.5)	1.00(ref)
Yes	303(13.1)	0.91(0.80–1.05)	198(8.5)	0.89(0.75–1.05)	264(11.4)	0.86(0.74–1.00)
Heart rate (beats/min)						
<80	1684(13.3)	1.00(ref)	1065(8.4)	1.00(ref)	1439(11.4)	1.00(ref)
80–100	668(16.2)	1.39(1.26–1.54)	542(13.1)	1.78(1.59–2.00)	728(17.6)	1.76(1.59–1.94)
≥100	78(18.6)	1.87(1.44–2.43)	72(17.1)	2.76(2.10–3.63)	95(22.6)	2.71(2.11–3.47)
MTHFR C677T Polymorphism						
CC	572(14.1)	1.00(ref)	373(9.2)	1.00(ref)	503(12.4)	1.00(ref)
CT	1210(14.1)	1.01(0.90–1.12)	837(9.7)	1.07(0.94–1.22)	1120(13.0)	1.07(0.95–1.20)
TT	648(14.3)	1.04(0.91–1.17)	469(10.3)	1.15(0.99–1.33)	639(14.1)	1.17(1.03–1.33)
Counties						
Ganyu(coastal)	938(12.5)	1.00(ref)	663(8.9)	1.00(ref)	900(12.0)	1.00(ref)
Donghai(inland)	1492(15.4)	1.32(1.20–1.45)	1016(10.5)	1.26(1.13–1.40)	1362(14.0)	1.20(1.09–1.32)
Living Standards						
Bad	261(13.7)	1.00(ref)	203(10.7)	1.00(ref)	277(14.6)	1.00(ref)
Medium	1867(14.0)	1.01(0.87–1.17)	1264(9.5)	0.85(0.72–1.00)	1698(12.8)	0.83(0.72–0.96)
Good	302(15.2)	1.08(0.89–1.30)	212(10.7)	0.93(0.75–1.16)	287(14.4)	0.90(0.74–1.09)
Education						
Illiterate	1616(14.3)	1.00(ref)	1140(10.1)	1.00(ref)	1530(13.5)	1.00(ref)
Primary level	325(12.9)	0.83(0.72–0.95)	237(9.4)	0.89(0.76–1.04)	317(12.6)	0.94(0.82–1.08)
Elementary or higher levels	489(14.5)	0.96(0.84–1.09)	302(9.0)	0.89(0.75–1.05)	415(12.3)	1.00(0.86–1.15)
Physical Activity						
Low	1021(14.7)	1.00(ref)	731(10.5)	1.00(ref)	1064(15.4)	1.00(ref)
Moderate	912(13.8)	0.94(0.85–1.04)	630(9.5)	0.94(0.83–1.05)	785(11.8)	0.82(0.73–0.91)
High	497(13.7)	0.92(0.81–1.04)	318(8.8)	0.84(0.72–0.98)	413(11.4)	0.76(0.67–0.87)

BMI = body mass index, FPG = fasting plasma glucose, HTN = hypertension, IFG = impaired fasting glucose, MTHFR =  methylenetetrahydrofolate reductase.

1 All variables were included in the same models;^ 2^474 subjects with antihypertensive treatment and controlled blood pressure were included; ^3^Obesity was defined as a BMI of ≥25kg/m^2^; ^4^Abdominal obesity was defined as a waist circumference ≥90 cm for men and ≥80 cm for women.

## Discussion

In our present study, from 2008–2009 the prevalence rates of total diabetes and IFG among Chinese hypertensive adults were 13.2%, and 14.1%, respectively. These figures are higher than a previous study of hypertensive rural Chinese conducted from 2004–2006 in Northern China (diabetes: 10.0%; IFG: 9.7%) [21], and also higher than those of Chinese adults aged 18 and over in the national nutrition and health survey in 2002 (diabetes: about 3.7% in Jiangsu province) [22]. The aging of the population, as well as dietary changes and decreasing levels of physical activity, with a consequent high prevalence of obesity and abdominal obesity [4], have probably contributed to the rapid increase in the prevalence of diabetes. However, the observed increase in the prevalence of diabetes could also be due to different population inclusion criteria, genetic and ethnic backgrounds, as well as differences in risk factor profiles across the regional areas.

Similar to previous studies [4], [23], [24], older age, obesity, abdominal obesity, a family history of diabetes, higher heart rate, and lower physical activity levels were important independent associated factors for diabetes in the present study. Most importantly, about 26.5% of the participants had a resting heart rate ≥80 beats/min. However, we observed an inverse relationship between smoking and diabetes, which was consistent with the Finnmark study with 12 years of follow-up [24], while others reported smoking and diabetes to be positively [25] or not related [4]. Furthermore, due to the high correlation (Pearson’s r = 0.78) between BMI and WC in our study, we also ran the other models: (1) overall obesity only; and (2) abdominal obesity only (Table 3). However, BMI and WC represent different aspects of body composition: BMI is a surrogate of overall adiposity while WC is a surrogate of central adiposity. Our results with both obesity and abdominal obesity in the model suggest an independent effect of obesity and abdominal obesity on diabetes (as did in a previous study [2]) and underscore the importance of controlling both obesity and abdominal obesity.

**Table 3:** The association between obesity and/or abdominal obesity and diabetes (total or previously undiagnosed) or IFG.

	*IFG*		*Previously* *undiagnosed diabetes*				*Total diabetes*			
	Prevalence,n (%)	Adjusted OR(95% CI)	Prevalence,n (%)		Adjusted OR(95% CI)		Prevalence,n (%)		Adjusted OR(95% CI)	
**Only Obesity in the model** 1										
Obesity^2^										
No	1040(13.3)	1.00(ref)	620(7.9)		1.00(ref)		864(11.1)		1.00(ref)	
Yes	1390(14.8)	1.20(1.06–1.37)	1059(11.3)		1.54(1.38–1.72)		1398(14.9)		1.42(1.29–1.56)	
**Only Abdominal Obesity in the model** 1										
Abdominal Obesity^3^										
No	920(13.3)	1.00(ref)	522(7.6)	1.00(ref)		677(9.8)		1.00(ref)		
Yes	1510(14.7)	1.25(1.13–1.37)	1157(11.3)	1.70(151–1.92)		1585(15.4)		1.70(1.53–1.89)		

1 Age, sex, cigarette smoking, alcohol drinking, antihypertensive treatment status, hypertension grades, heart rate, MTHFR C677T polymorphism, geographic region, standard of living, education level, physical activity level and family history of hypertension, diabetes, CHD or stroke were also adjusted in the models. ^2^Obesity was defined as a BMI of ≥25kg/m^2^. ^3^Abdominal obesity was defined as a waist circumference ≥90 cm for men and ≥80 cm for women.

In the current study, there was a non-significant, positive association between hypertension grades and previously undiagnosed diabetes. However, although participants with antihypertensive treatment had lower SBP (mean(SD): 168.0(22.7) versus 169.1(19.0) mmHg, *P* = 0.001) and DBP (94.9(12.3) versus 95.7(11.5) mmHg, *P*<0.001) than those without antihypertensive treatment, participants with antihypertensive treatment had a higher prevalence of total diabetes and previously undiagnosed diabetes compared to those without antihypertensive treatment. Consistently, antihypertensive treatment was associated with a two- to three-fold increased risk of diabetes in the 12-year follow-up Finnmark study in Norway [24]. Mozaffarian D et al. also reported that the use of beta-blockers and diuretics were independent risk factors for new-onset diabetes or IFG in a prospective study with 8291 Italian patients with myocardial infarction (mean follow-up 3.2 years) [23]. However, given the cross-sectional design of our study, the causal relationship between antihypertensive treatment and diabetes is still inconclusive and needs to be further investigated.

Benes P et al. first reported that the C allele of the MTHFR C677T polymorphism was associated with diabetes in women in the Czech population [26]. However, other small sample studies have reported a positive association between T allele and diabetes (n = 336) [27], or no association between MTHFR C677T polymorphism and diabetes (n = 118) [28]. In our study, participants with MTHFR 677 TT genotype had a significantly higher prevalence of total diabetes. The frequency of MTHFR 677 TT genotype was about 26.4% in this population. Our results further suggest that MTHFR 677 TT genotype may be a useful marker for the early detection of a population at high risk for diabetes among Chinese hypertensive adults. However, further studies are necessary to confirm our results and elucidate the underlying mechanism of the MTHFR C677T polymorphism’s involvement in the development of diabetes.

Most interestingly, residents who lived inland (versus coastal) had a significantly higher prevalence of IFG and diabetes even in the fully-adjusted models. The regional difference may partly be explained by a higher intake of n-3 fatty acids [29], [30]. However, we did not have detailed information about seafood intake in this study. Further identification of the mechanism may help to better understand the etiology of diabetes in the future, and may lead to improved strategies for early prevention, identification, and treatment.

We excluded participants with known CVD, cancer, and dyslipidemia from our analyses to permit us to exclude for the possibility of confounding our results due to concomitant diseases or medications. Our study population was not a representative sample. Caution is needed in generalizing our findings from this hypertensive Chinese population to other populations. Our study was cross-sectional. Therefore, the temporal nature of the association between the studied associated factors and diabetes cannot be established from our study. Furthermore, we did not collect information from rural migrants, which may possibly have produced some bias in our results [31]. The other limitation of our study is that a 2-hour oral glucose tolerance test (OGTT) was not conducted, which likely made us underestimate the prevalence of diabetes. However, although the 75-g OGTT is more sensitive than the FPG in diagnosing diabetes, it is difficult to perform in practice, particularly in rural China. The foundation of prevention and treatment for IFG and diabetes is lifestyle modification. In our study, compared to participants with previously undiagnosed diabetes, participants with previously diagnosed diabetes were less likely to smoke cigarettes and drink alcohol, and more likely to take antihypertensive treatment, which suggests an improvement in lifestyle and treatment attitude after the identification of diabetes in Chinese hypertensive adults. Furthermore, even if only based on the FPG evaluation, about 74.2% of the participants with diabetes had not previously been diagnosed. This is similar to findings from a previous study in China [3] in which 76% of diabetes was undiagnosed. So, considering the ease of use, acceptability to patients, and lower cost, we suggest that the FPG should be the preferred screening test in Chinese rural areas.

In conclusion, our study found a high prevalence of diabetes in Chinese hypertensive adults. Furthermore, about three out of every four diabetic adults were undiagnosed. Our results suggest that population-level measures aimed at the prevention, identification (even if only based on the FPG evaluation), and treatment of diabetes should be urgently taken to overcome the diabetes epidemic in Chinese hypertensive adults.
